# Early-line immunotherapy in multiple myeloma: reshaping the treatment paradigm

**DOI:** 10.3389/fimmu.2026.1981988

**Published:** 2026-10-30

**Authors:** Rida Darji, Curtis Marcoux, Mahmoud Gaballa, Aimaz Afrough, Meera Mohan, Krina K. Patel, Oren Pasvolsky

**Affiliations:** 1 University of South Florida Morsani College of Medicine, Tampa, FL, United States; 2 Division of Hematology, Dalhousie University, Halifax, NS, Canada; 3 Department of Lymphoma and Myeloma, The University of Texas MD Anderson Cancer Center, Houston, TX, United States; 4 Hematologic Malignancies and Cellular Therapy Program, Simmons Comprehensive Cancer Center, University of Texas (UT) Southwestern Medical Center, Dallas, TX, United States

**Keywords:** bispecific antibodies, CAR T, cell therapy, first line, trials

## Abstract

Multiple myeloma (MM) has traditionally been considered to be incurable, despite increasingly effective induction, transplantation, and maintenance strategies. Chimeric antigen receptor (CAR) T-cell therapies and T-cell–redirecting bispecific antibodies have produced deep and durable responses in heavily pretreated disease and are now moving into earlier lines of therapy. The biologic rationale for earlier use includes the potential for greater T-cell fitness, a more permissive bone marrow immune microenvironment, a lower burden of treatment-resistant disease, and the need to deliver effective therapy before attrition prevents patients from receiving effective treatments. Randomized trials have established the superiority of B-cell maturation antigen (BCMA)-directed CAR T-cell therapy and BCMA- or G protein–coupled receptor class C group 5 member D (GPRC5D)-directed bispecific regimens over conventional therapies in early relapse. In contrast, frontline use remains investigational, with predominantly small, nonrandomized studies and limited follow-up. Ongoing studies are evaluating these approaches as induction, consolidation, maintenance, and measurable residual disease (MRD)–directed therapy, as well as potential alternatives to autologous stem cell transplantation. However, moving T-cell–redirecting immunotherapy earlier introduces important biological and clinical challenges, including sustained immune and antigen engagement, antigen escape and distinct toxicities. Optimal sequencing across targets and treatment modalities, as well as the optimal duration of therapy, remain areas of active investigation. The high efficacy of modern anti-CD38–based quadruplets and transplantation also establishes a demanding benchmark against which early-line immunotherapy must be assessed. In this review, we examine the immunological and clinical rationale for earlier treatment, summarize emerging evidence for CAR T-cell therapies and bispecific antibodies in early-relapsed and newly diagnosed myeloma, and discuss their evolving relationship with autologous transplantation. We further highlight the need for prospective sequencing studies, sustained MRD endpoints and fixed-duration/response-adapted strategies to determine whether earlier immunotherapy can provide durable disease control while reducing cumulative treatment exposure.

## 1 Introduction

Multiple myeloma (MM) has historically been treated through sequential lines of therapy, with each relapse typically associated with shorter response duration, increasing treatment resistance, and cumulative immune dysfunction. The routine use of proteasome inhibitors (PIs), immunomodulatory drugs (IMiDs), anti-CD38 monoclonal antibodies, and autologous stem cell transplantation (ASCT) has substantially improved survival (1). However, myeloma remains incurable for most patients, and many patients never receive later-line therapy because of various reasons including disease progression or death, treatment-related toxicities or other medical comorbidities, frailty, as well as access barriers, that could be financial, logistical and related to healthcare access (2, 3).

Immunotherapy has recently become one of the most active areas of myeloma therapeutics. T-cell directed therapies such as chimeric antigen receptor (CAR) T-cell therapies and bispecific antibodies (bsAb) have produced deep responses in patients with relapsed or refractory MM, including those previously exposed to PIs, IMiDs, and anti-CD38 antibodies (4). These results have raised the question of whether immune-based therapies should remain reserved for later relapse or incorporated earlier in the treatment course, when patients may be less heavily pretreated and have more preserved immune function.

This review focuses on the movement of immunotherapy into earlier lines of MM treatment. We first summarize the biological and clinical rationale for earlier use, then review emerging data for CAR T-cell therapy and bispecific antibodies in early relapse and frontline settings. We also discuss the evolving role of ASCT, unresolved sequencing questions and the possibility that effective immune-based therapy may eventually facilitate fixed-duration or measurable residual disease (MRD)-adapted treatment strategies.

### 1.1 Literature identification and selection

This narrative review draws on relevant publications identified through PubMed and ClinicalTrials.gov, supplemented by abstracts from American Society of Hematology (ASH), American Society of Clinical Oncology (ASCO), European Hematology Association (EHA), and International Myeloma Society (IMS) meetings. We focused on the biologic rationale and clinical evidence for earlier-line immunotherapy in multiple myeloma, distinguishing randomized evidence from single-arm studies, preliminary conference reports and ongoing trials. No systematic literature search or formal assessment of study quality was performed.

## 2 Rationale for early-line immunotherapy in myeloma

The current frontline treatment paradigm for newly diagnosed multiple myeloma is built on combination induction therapy, increasingly with quadruplet regimens, followed by consolidation and maintenance strategies that are largely determined by ASCT eligibility. Anti-CD38 monoclonal antibodies, specifically daratumumab and isatuximab, are now established parts of frontline triplet and quadruplet regimens in both transplant eligible and transplant-ineligible patients (5–8). Their successful incorporation into upfront therapy has shown that immune-based treatment can be safely and effectively moved earlier in the disease course. Whether this principle extends to novel immunotherapies, including CAR T-cell therapy and bispecific antibodies, is now a central question in myeloma care.

The rationale for earlier use of these therapies is based on several observations, including preservation of immune effector function, a potentially more favorable tumor microenvironment, and the understanding that at least for some patients there might not be a later chance at achieving remission and it might be best to use potent immunotherapy strategies early on.

### 2.1 T-cell fitness and the myeloma microenvironment

A central rationale for earlier use of CAR T-cell therapy and bispecific antibodies is that these treatments depend on functional T cells. CAR T-cell therapy requires collection, engineering, expansion, and persistence of patient-derived T cells, while bispecific antibodies rely on endogenous T cells to engage and kill malignant plasma cells. As a result, the number, phenotype and functional capacity of T cells can directly influence treatment efficacy (9).

This broader principle is also supported by data from anti-CD38 antibody-based therapy. An analysis from the CASSIOPEIA trial demonstrated that a lower proportion of CD16+ cytotoxic NK cells in the bone marrow at diagnosis was associated with reduced likelihood of achieving MRD negativity after daratumumab-based therapy, but not in the control arm without daratumumab, linking baseline NK-cell composition with responses to anti-CD38 immunotherapy (10). Similarly, in newly diagnosed patients treated with daratumumab, carfilzomib, lenalidomide, and dexamethasone (D-KRd) in the MANHATTAN trial, a higher baseline proportion of bone marrow NK cells was associated with sustained MRD negativity (11). Although these studies did not directly compare newly diagnosed and relapsed populations, they support the broader concept that baseline immune state is associated with the depth and durability of response to immune based therapy.

The relationship between immune fitness and treatment response is particularly relevant for T-cell–redirecting therapy. In correlative analyses from the MajesTEC-1 trial, relapsed/refractory multiple myeloma (RRMM) patients receiving the BCMA-directed bispecific antibody teclistamab, had better outcomes when they had higher baseline peripheral T-cell counts, lower proportions of immunosuppressive regulatory T-cells (Tregs) and fewer T cells expressing exhaustion-associated markers (12). In a separate single-cell analysis of bone marrow T cells from patients receiving the BCMA-directed bispecific antibody elranatamab, Friedrich et al. found that response was associated with clonal expansion of effector CD8+ T cells, whereas a higher abundance of exhausted-like CD8+ T-cell clones was associated with treatment failure (13). Ex vivo studies have further shown that bispecific antibody–mediated myeloma cell lysis is impaired in samples with high Treg numbers and low T-cell–to–myeloma cell ratios, and that continuous bispecific antibody exposure drives acquired T-cell exhaustion with reduced proliferative capacity and cytokine secretion (14). Together, these data suggest that T-cell state may influence response to T-cell–engaging therapy.

This is relevant to treatment timing because the T-cell compartment changes throughout the disease course and with treatment exposure. In patients undergoing ASCT, those who relapsed within 12 months had higher proportions of T cells with senescence- or exhaustion-associated markers compared with patients who did not relapse early. These differences were already detectable at 3 months, even before clinical relapse was apparent (15). Additional work has implicated T-cell immunosenescence as a contributor to impaired anti-myeloma immunity and disease progression (16). Recent mass cytometry and TCR sequencing data from 45 myeloma patients have shown that those with short (<3 years) overall survival (OS) had significantly fewer T cells, increased Tregs, and more activated and exhausted CD8+ T cells at relapse compared with long-term survivors, and that higher PD-1 expression on CD8+ T cells at diagnosis correlated with shorter overall survival (17).

T-cell dysfunction in myeloma is heterogeneous and varies by disease stage and prior therapy. Earlier studies characterized exhaustion and senescence-associated phenotypes in relapsed disease (15, 16), but more recent profiling work has challenged the classic exhaustion model in newly diagnosed myeloma. Shasha et al. found no evidence of functionally exhausted CD8+ T cells or antigen-driven clonal expansion across longitudinal samples from diagnosis through maintenance therapy (18), and Foster et al. demonstrated that T-cell populations previously labeled as exhausted may instead reflect antigen-driven terminal memory differentiation (19). This distinction is relevant because it may help explain why checkpoint inhibitors have had limited efficacy in myeloma, while T-cell engaging therapies that activate or supply functional effector cells, including bispecific antibodies and CAR T, have shown more clinical activity (20). These observations provide a rationale for earlier T-cell-redirecting therapy, although most are correlative and based on relapsed disease. Establishing that preserved immune fitness itself drives better outcomes rather than simply reflecting less pretreated disease will require frontline trials that pair long-term PFS and OS with baseline T-cell profiling.

The bone marrow microenvironment may also be more favorable earlier in the disease course. Spatial analyses using immunohistochemistry have shown that CD8+ T cells are more frequently located near plasma cells in newly diagnosed myeloma than in relapsed/refractory disease, despite similar plasma cell burden between cohorts (21). While this does not establish that T-cell redirecting therapy will be more effective upfront, it adds to a growing body of evidence that the immune microenvironment in earlier disease may be more permissive to immunotherapy. The cumulative effects of prior therapy, evolving immune fitness and microenvironmental changes all contribute to the context in which immunotherapy is delivered.

### 2.2 Treatment attrition and access to later-line therapy

A separate but important rationale for earlier immunotherapy is that many patients with myeloma are not able to receive multiple subsequent lines of therapy. Real-world studies consistently show that attrition increases across successive lines, particularly among older, frailer, and non-transplant patients. In a large US analysis of over 24, 000 patients with NDMM, only 43% of non-transplant patients and 79% of transplant patients received more than one line of therapy, and only 8% and 22%, respectively, reached fifth-line therapy (2). A Canadian Myeloma Research Group analysis of 5, 548 patients further demonstrated that attrition rates increase with each line and are substantially higher in non-transplant patients (19% after line 1, 26% after line 2, 40% after line 3), with death as the dominant contributor (22). More recent data from the Flatiron database specifically in lenalidomide-refractory patients, showed a cumulative attrition rate of 85% from second to fifth line of therapy (23). A 40-year retrospective analysis similarly found that despite improvements over time, 46.9% of patients over age 80 were unable to proceed beyond first-line therapy. While attrition rates after each line of therapy (1–4) decreased in the most recent decade studied (2010–2020) and OS improved across all lines of therapy, progression-free survival (PFS) was only significantly improved after the first line of therapy (3). These data underscore that reserving the most effective therapies for later lines effectively denies them to a substantial proportion of patients.

Overall, the rationale for early-line immunotherapy is that these agents may be most effective, and most deliverable, before cumulative treatment exposure, immune dysfunction, and clinical attrition limit their benefit. Correlative immune data, studies of the myeloma microenvironment, and real-world attrition data support further evaluation in earlier relapse and frontline studies.

## 3 Evidence and ongoing trials with early-line immunotherapy in multiple myeloma

### 3.1 CAR T-cell therapies

#### 3.1.1 From late-line therapy to earlier relapse

Chimeric antigen receptor (CAR) T-cell therapy has become one of the most active immune-based treatment strategies in relapsed/refractory multiple myeloma. Current approved products in myeloma target the B-cell maturation antigen (BCMA), a plasma-cell antigen highly expressed on malignant plasma cells. Idecabtagene vicleucel (ide-cel) and ciltacabtagene autoleucel (cilta-cel) were initially developed in heavily pretreated patients, but randomized phase 3 trials have now moved CAR T-cell therapy into earlier relapse.

The initial approvals of ide-cel and cilta-cel were based on single-arm studies in heavily pretreated disease. In the phase 2 KarMMa trial, ide-cel had an overall response rate (ORR) of 73%, with complete response (CR) or better in 33%, and a median PFS of 8.8 months in patients who had received a median of six prior lines of therapy and were exposed to a PI, IMiD, and anti-CD38 monoclonal antibody (triple class exposure) (24). Similarly, CARTITUDE-1 established cilta-cel as highly active in relapsed/refractory myeloma, with an ORR of 98%, stringent CR rate of 83%, and median PFS of 34.9 months in patients who were triple-class exposed and had received a median of six prior lines of therapy (25). With longer follow-up, CARTITUDE-1 also showed that a subset of patients can achieve prolonged treatment-free disease control. At a median follow-up of 61.3 months, median overall survival was 60.7 months, and 32 of 97 patients remained alive and progression-free for at least 5 years after a single cilta-cel infusion, without maintenance or subsequent therapy (26). Although this represents a selected subset of patients, the durability of these responses is an important reason CAR T-cell therapy is now being evaluated earlier in the disease course.

The first randomized evidence for earlier use of CAR T-cell therapy came from KarMMa-3 and CARTITUDE-4. KarMMa-3 compared ide-cel with physician’s choice of five standard regimens, including daratumumab-, carfilzomib-, ixazomib-, and elotuzumab-based combinations, in patients with triple-class exposed relapsed/refractory myeloma after two to four prior lines of therapy, with most patients having triple-class refractory disease. Ide-cel significantly improved median PFS compared with standard therapy (13.3 vs. 4.4 months) with higher overall response and complete response rates (27). Overall survival was not improved in the primary analysis. However, results may have been confounded by the high crossover rate (56%) from the standard of care arm to ide-cel following disease progression (28).

CARTITUDE-4 evaluated cilta-cel in patients with lenalidomide-refractory myeloma after one to three prior lines of therapy. Patients were required to be exposed to both a PI and an IMiD, but prior anti-CD38 exposure was not required. At the primary analysis, cilta-cel significantly improved PFS compared with physician’s choice of pomalidomide, bortezomib, dexamethasone (PVd) or daratumumab, pomalidomide, dexamethasone (DPd). Median PFS was not reached with cilta-cel versus 11.8 months with standard therapy, with higher rates of ORR, CR or better, and MRD negativity (10^-5^: 60.6% vs. 15.6%) (29). With longer follow-up, CARTITUDE-4 demonstrated an overall survival advantage for cilta-cel, with estimated 30-month OS of 76.4% versus 63.8% compared with standard therapy (HR 0.55, p=0.0009) (30). A real-world study comparing cilta-cel as an early-line therapy (1–3 prior lines) to a late-line therapy (>3 prior lines) reported 12-month PFS of 79% and 70%, respectively, with numerically better outcomes in earlier-line use (31).

Taken together, KarMMa-3, and CARTITUDE-4 established CAR T-cell therapy as an option at earlier relapse, but they do not define how ide-cel and cilta-cel should be selected relative to each other. The trials addressed different clinical questions, with KarMMa-3 focused on a later-line, triple-class exposed population and CARTITUDE-4 focused on lenalidomide-refractory disease earlier in relapse. Although no prospective trial has directly compared ide-cel and cilta-cel, the most informative comparative data comes from a 19-institution retrospective analysis by the US Multiple Myeloma Immunotherapy Consortium of patients treated with standard-of-care CAR T-cell therapy. In that study, cilta-cel was associated with higher rates of CR or better, longer PFS, and longer OS compared with ide-cel after adjustment for baseline differences, but also higher rates of grade 3 or higher cytokine release syndrome, infections, and delayed neurotoxicity (32). In practice, selection is driven by indication, access, comorbidities, toxicity profile, and institutional experience.

#### 3.1.2 Current studies investigating BCMA CAR T-cell therapy expansion to frontline

With the success of CAR T-cell therapy in earlier relapse, several studies are now evaluating BCMA-directed CAR T-cell therapy in newly diagnosed patients. CARTITUDE-5 (NCT04923893) is comparing VRd induction followed by a single cilta-cel infusion with VRd induction followed by Rd maintenance in newly diagnosed patients not planned for ASCT as part of initial therapy. The aim is to determine whether CAR T-cell therapy can provide a finite consolidation strategy rather than ongoing maintenance (33). CARTITUDE-6 (NCT05257083) is a phase 3 clinical trial evaluating cilta-cel in transplant-eligible patients after DVRd induction, with cilta-cel compared against ASCT-based consolidation and lenalidomide maintenance planned in both arms for 2 years (34). KarMMa-4 (NCT04196491) is evaluating ide-cel after induction in transplant-eligible patients with high-risk (R-ISS stage III) newly diagnosed myeloma (35).

Several smaller studies have reported early results with investigational BCMA-directed CAR T-cell products in newly diagnosed myeloma. CAREMM-001 (NCT05860036) evaluated BCMA-directed CAR T-cell therapy in newly diagnosed patients who were ineligible for or not proceeding to ASCT and reported high rates of MRD negativity and deepening responses over time (36). A conference abstract reporting early results from FUMANBA-2 (NCT05181501) evaluating equecabtagene autoleucel in transplant-ineligible patients with high-risk newly diagnosed myeloma demonstrated high rates of stringent CR and early MRD negativity (37). These studies provide early evidence that frontline CAR T-cell therapy can induce deep responses, but remain limited by small sample size, short follow-up, single-arm design, and use of investigational products. As discussed in Section 3.2.2, cross-study comparison of these MRD and response rates is further limited by differences in assay sensitivity, timing of assessment, and the denominator used to report MRD rates. Whether these findings translate into broader use of CAR T-cell therapy in newly diagnosed myeloma will depend on results from ongoing phase 3 frontline trials.

CAR T-cell therapy is also being explored even earlier on in the spectrum of plasma cell disorders, including high-risk smoldering myeloma. In the CAR-PRISM study of cilta-cel, all 20 treated patients achieved MRD negativity after CAR T-cell therapy without induction or bridging therapy (38). Whether CAR T-cell therapy in this setting prevents clinically meaningful myeloma-related complications or improves long-term outcomes remains to be determined, and will need to be weighed against toxicity, cost, and the risk of overtreatment.

#### 3.1.3 Novel CAR T-cell therapy targets

As BCMA-directed therapies, including CAR T-cell therapy and bispecific antibodies, move into earlier lines, alternative and dual-target CAR T-cell approaches are being developed to expand treatment options, particularly after prior BCMA exposure. Among non-BCMA targets, G protein–coupled receptor class C group 5 member D (GPRC5D) has attracted the most attention, supported by the clinical activity of GPRC5D-directed bispecific antibodies and early CAR T-cell studies in the relapsed/refractory setting. In a conference abstract of a phase 1 study of the anti-GPRC5D CAR T-cell therapy arlocabtagene autoleucel (arlo-cel) in patients with relapsed/refractory myeloma after one to three prior regimens, the ORR was 96% and the CR rate was 67% among 24 efficacy-evaluable patients after a median follow-up of 18.3 months (39). Arlo-cel is now being compared with standard regimens in the phase 3 QUINTESSENTIAL-2 trial in lenalidomide-exposed myeloma after one to three prior lines of therapy (NCT06615479) (40). GPRC5D targeting also introduces distinct toxicities, including taste, skin, nail, and mucosal effects reported particularly with bispecific antibodies (41), and cerebellar toxicity reported with GPRC5D-directed CAR T-cell therapy (42).

Dual-target CAR T-cell approaches are also being developed, including BCMA/GPRC5D and BCMA/CD19 constructs. The rationale is to reduce relapse related to antigen escape or clonal heterogeneity, although it remains unproven that dual-target CAR T-cell therapy improves durability or safety compared with sequential single-target approaches (43). A phase I study of concurrent BCMA- and GPRC5D-directed CAR T-cell products showed clinical activity, but correlative analyses identified persistent antigen escape and reduced BCMA-CAR T-cell expansion with dual targeting (44). Early frontline activity has also been reported with AZD0120, formerly GC012F, a BCMA/CD19 dual-target product (45). Selected investigational CAR T-cell targets in development for early-line treatment of MM are summarized in **Table 1**.

**Table 1:** Selected investigational CAR T-cell therapy strategies in early-line multiple myeloma.

Clinical setting	Trial	Product/target	Phase and design	Key population	Treatment strategy/comparator	Primary endpoints and available results
Frontline induction/consolidation	**NCT05840107**	AZD0120 (formerly GC012F); BCMA/CD19 dual-target CAR T	Early phase; open-label; single-arm	NDMM, transplant-ineligible patients aged ≥70	AZD0120 following VRD induction	Primary endpoints: ORR, PFS, CR and stringent CR rates, MRD negativity rate, duration of response, AEs Available results: Of the 8 patients infused, 50% were defined as frail. At a median follow-up of 9.8 months, hematologic toxicities were the most common ≥grade 3 adverse events. All patients achieved stringent CR. All patients achieved MRD negativity (10^−6^) at 1 month (45).
Frontline induction/consolidation	**NCT04935580**	AZD0120 (formerly GC012F); BCMA/CD19 dual-target CAR T	Early phase; open-label; single-arm	NDMM, high-risk, transplant-eligible	AZD0120 following bortezomib-based induction	Primary endpoints: ORR, PFS, MRD negativity rate (10^−5^ to 10^−4^), duration of response, AEs Available results: All patients (n=22) responded (ORR 100%) across all tested dose levels. All patients achieved MRD negativity (10^−6^). Stringent CR rate was 95% (46).
Frontline and early relapse	**DURGA-2** **NCT07073547**	AZD0120 (formerly GC012F); BCMA/CD19 dual-target CAR T	Phase I; modular; multicenter; open-label	NDMM and early-relapsed or primary-refractory MM after 1–2 prior lines	AZD0120 ± maintenance strategies in NDMM modules	Primary endpoints: Incidence and severity of AEs, dose-limiting toxicities Available results: Ongoing study; data not yet available
Frontline induction/consolidation	**CARTITUDE-10** **NCT07149857**	Ciltacabtagene autoleucel (cilta-cel); BCMA CAR T	Phase II; multicohort	NDMM, ineligible for high-dose chemotherapy with transplant	Cilta-cel following lymphodepletion strategies containing cyclophosphamide alone or cyclophosphamide with fludarabine	Primary endpoints: MRD negative (10^−5^) CR rates Available results: Ongoing study; data not yet available
Post-induction/ early consolidation	**NCT03549442**	BCMA CAR T with or without humanized CD19 CAR T (huCART19)	Phase I; open-label	High-risk MM responding inadequately or progressing during first- or second-line therapy	BCMA CAR T alone or combined/ sequenced with huCART19	Primary endpoints: AE reporting Available results: Results reported on donor-specific allo-antibody reduction; myeloma efficacy data is limited/preliminary
Early relapse	**QUINTESSENTIAL-2** **NCT06615479**	Arlocabtagene autoleucel (arlo-cel, BMS-986393); GPRC5D CAR T	Phase III; randomized; open-label; multicenter	RRMM in earlier-line setting	Arlo-cel versus standard-of-care regimens	Primary endpoints: PFS and MRD negativity in CR Available results: Ongoing study; data not yet available (40)
Early relapse	**NCT06121843**	Arlocabtagene autoleucel (arlo-cel, BMS-986393); GPRC5D CAR T	Phase I; multicenter; open-label	RRMM; expansion cohorts include earlier relapse depending on the arm	Arlo-cel combinations with alnuctamab (IgG1 bispecific), elranatamab (BCMA bispecific), mezigdomide (CELMoD), iberdomide (CELMoD)	Primary endpoints: Incidence and severity of AEs, incidence of AEs of special interest, incidence of AEs resulting in discontinuation, number of deaths, establishing a recommended phase 2 dose Available results: Ongoing study; data not yet available
Early relapse	**DURGA-4** **NCT07391657**	AZD0120 (formerly GC012F); BCMA/CD19 dual-target CAR T	Phase III; randomized; multicenter; open-label	RRMM after 1–3 prior lines of therapy	AZD0120 vs. investigator-selected DKd, DPd, PVd or Kd	Primary endpoints: PFS, MRD negative CR rate at 9 months Available results: Ongoing study; data not yet available
Early relapse	**iMMagine-3** **NCT06413498**	Anitocabtagene autoleucel (anito-cel); BCMA CAR T with a D-domain binder	Phase III; randomized; open-label	RRMM after 1–3 prior lines of therapy, including prior anti-CD38 antibody and IMiD exposure	Anito-cel versus standard-of-care therapy	Primary endpoints: PFS, MRD negative (10^−5^) CR rates at 9 months Available results: Ongoing study; data not yet available (47)

AE, adverse event; ASCT, autologous stem-cell transplantation; BCMA, B-cell maturation antigen; CELMoD, cereblon E3 ligase modulator drug; CD3, cluster of differentiation 3; CR, complete response; DKd, daratumumab-carfilzomib-dexamethasone; DPd, daratumumab-pomalidomide-dexamethasone; D-VRd, daratumumab-bortezomib-lenalidomide-dexamethasone; GPRC5D, G protein-coupled receptor class C; IMiD, immunomodulatory drug; Kd, carfilzomib-dexamethasone; MM, multiple myeloma; MRD, measurable residual disease; NDMM, newly diagnosed multiple myeloma; ORR, overall response rate; PFS, progression free survival; PVd, pomalidomide-bortezomib-dexamethasone; RRMM, relapsed/refractory multiple myeloma.

Bolded items indicate clinical trial names and their corresponding NCT identification numbers.

#### 3.1.4 Potential challenges to expanding CAR T-cell therapy to frontline use

##### 3.1.4.1 What is the threshold for a benefit to be considered significant?

Moving CAR T-cell therapy earlier in myeloma raises questions beyond efficacy observed in relapsed disease. In newly diagnosed myeloma, CAR T-cell therapy must be evaluated against established frontline strategies, including anti-CD38-based quadruplet induction, ASCT and maintenance. Particularly for standard-risk patients, the prolonged disease control achieved with current treatment raises the threshold for accepting additional toxicity or treatment burden simply to increase early MRD-negativity rates. Sustained MRD negativity would be more compelling if accompanied by longer PFS or evidence that treatment can be safely discontinued. Even without an OS advantage, comparable disease control with substantially less treatment burden could justify earlier use, provided this is not offset by late toxicity. Prolonged time off treatment is therefore an important potential benefit of both CAR T-cell therapy and fixed-duration bispecific approaches. However, these strategies should be compared with contemporary treatment, including approaches that shorten maintenance, rather than assuming that the alternative is indefinite therapy. These comparisons should therefore report time off all therapy, late toxicity, and quality of life as prespecified endpoints, not only PFS.

Sequencing will also become increasingly important as BCMA-directed therapies move earlier in treatment. Use of BCMA-directed CAR T-cell therapy upfront may affect subsequent responses to BCMA-directed bispecific antibodies, while prior bispecific antibody exposure may affect later CAR T-cell outcomes. These questions are particularly relevant if both CAR T-cell therapy and bispecific antibodies are being evaluated in earlier lines using the same target. However, the clinical magnitude of these sequencing effects in less heavily treated patients remains uncertain. Much of the current evidence comes from later-line cohorts, where prior therapy, disease biology, and T-cell dysfunction may not fully reflect the frontline setting.

##### 3.1.4.2 Can we deliver CAR T-cell therapy early enough for all patients?

Beyond sequencing, delivery of CAR T-cell therapy remains a major consideration. CAR T-cell therapy requires specialized centers, most of which are tied to large academic institutions. Unlike most systemic therapies used in frontline myeloma, CAR T-cell therapy requires coordination across referral, leukapheresis, manufacturing, lymphodepletion, acute toxicity monitoring, and long-term follow-up. These steps create challenges related to timing, caregiver support, proximity to a cellular therapy center, socioeconomic factors, and funding. These access issues are already evident in the relapsed/refractory setting, and would become more pronounced if CAR T-cell therapy were used in larger frontline populations. The potential quality-of-life benefit of a one-time therapy therefore depends on whether patients can reach infusion before disease progression, clinical decline, or loss of eligibility. When analyzing global disparities limiting CAR T-cell therapy access, a study looking at fourteen G20 countries showed that only 48% of FDA CAR T-cell therapy product-indication pairs for hematologic malignancies are publicly reimbursed and when they are, patients typically have to wait more than one and a half years after FDA approval before their therapy is covered by their health system (48). As such, even when there is FDA or equivalent CAR T-cell therapy approval and an infrastructure in place to administer CAR T-cell therapy, many patients are still unable to access care globally.

When CAR T-cell therapy is accessible for patients, there are several barriers that can prevent patients from receiving CAR T-cell therapy even after completing leukapheresis. Product availability is an important limitation to consider, especially as CAR T-cell therapy manufacturers have often been unable to keep up with current demand. An early multi-institutional survey found that there was a median of one manufacturing spot allocated per month, resulting in a median waitlist of 20 patients per center and a median wait time of 6 months before getting to leukapheresis (49). Even after leukapheresis, there was an average time spent waiting for approved products of 13–54 days according to a separate review. Up to 31% of patients who underwent leukapheresis never received their CAR T-cell therapy infusion due to, most commonly, disease progression during the manufacturing window or manufacturing failure during wait times (50). If CAR T-cell therapy moves into frontline myeloma, the eligible population would expand substantially, making manufacturing capacity, referral pathways, and slot allocation challenges even more relevant.

While there has been improvement in the percentage of patients who end up receiving CAR T-cell therapy after leukapheresis in some more recent studies, this data appears to be uneven in the emerging literature. In the CARTITUDE studies, non-infusion rates among leukapharesed patients improved over time from 14.2% in CARTITUDE-1 to 5.8% in CARTITUDE-4, with disease progression during bridging therapy or lymphodepletion being the most common cause for pre-CAR T-cell infusion attrition in CARTITUDE-4 (29, 51). In a retrospective study from 2024 reviewing outcomes of cilta-cel in the standard-of-care setting, only 7.5% of patients did not receive cilta-cel after leukapheresis (52). However, a 2026 real-world analysis of a claims database demonstrated that over half of the patients who received leukapheresis did not receive CAR T-cell therapy, indicating that pre-CAR T-cell attrition may still be a persistent factor affecting effective care in the real-world setting (53).

There are several strategies that are currently being employed to reduce the risk of disease progression during the manufacturing period, such as bridging therapy after CAR T-cell apheresis, which the International Myeloma Working Group (IMWG) Immunotherapy Committee currently recommends considering in patients with high-disease burden or at a higher risk of mortality within the next 4–6 weeks (54). With the development of more rapid CAR T-cell manufacturing platforms, there is potential for significantly shorter vein-to-vein times, such as with the rapid manufacturing seen with the FasTCAR platform that manufactures the novel AZD0120 CAR product (55). Further investigation into off-the-shelf allogeneic CAR T-cell therapy, which would derive CAR T-cells from healthy donors rather than individual patients and would allow treatment to be manufactured in batches, may alleviate manufacturing bottlenecks, manufacturing failure and development cost. While all current allogeneic CAR T-cell products remain investigational, this could potentially expand access if efficacy, persistence, and safety are comparable to autologous CAR T-cell therapy (56).

##### 3.1.4.3 Can we accept the long-term adverse effects?

Safety also needs to be considered differently in newly diagnosed disease. Cytokine release syndrome (CRS) and immune effector cell-associated neurotoxicity syndrome (ICANS) are usually early and manageable in experienced centers, but CAR T-cell therapy can also be associated with delayed or prolonged toxicity, including persistent cytopenias, infections, delayed neurologic events, immune effector cell-associated enterocolitis and secondary malignancies (50). In a review across hematologic malignancies, infections accounted for more than half of non-relapse deaths after CAR T-cell therapy, while CRS, ICANS/neurotoxicity, hemophagocytic lymphohistiocytosis accounted for only 11.5% (57). Secondary malignancies and late hematotoxicity are additional concerns, although estimates from heavily pretreated populations may not reflect those of newly diagnosed patients or those at early relapse (58, 59). In newly diagnosed patients who may achieve prolonged disease control with established treatment, persistent immune suppression or late toxicity could offset the benefit of a deeper initial response. Earlier use therefore requires more than manageable acute toxicity. Frontline comparisons will need to establish how long cytopenias and hypogammaglobulinemia persist, whether patients remain dependent on immunoglobulin replacement or susceptible to serious infections, and the longer-term risk of secondary malignancies.

##### 3.1.4.4 Who truly requires early CAR T-cell therapy?

The potential benefit of early CAR T-cell therapy is also likely to vary by disease risk and response to initial treatment. High-risk cytogenetics provide a rationale for improving frontline disease control, whereas primary resistance or early progression suggests that an alternative treatment mechanism may be needed (60). It is not yet known whether earlier intervention can overcome high-risk biology and the high-risk subgroups of KarMMa-4 and CARTITUDE-6 may help address this. Persistent MRD in an otherwise responding patient is a less clear reason to change treatment. Its significance depends on when it is assessed, whether it persists through subsequent therapy and the underlying cytogenetic risk (61, 62). Extramedullary disease presents a different challenge, since deep marrow responses may not reflect control of disease outside the marrow. These distinctions should guide patient selection in frontline trials, rather than treating high-risk cytogenetics, early resistance and persistent MRD as interchangeable reasons for early intervention.

Selected older and frail patients may benefit from CAR T-cell therapy, although they may be at increased risk of both developing ICANS and experiencing greater ICANS severity. While some studies have shown no significant difference in long-term outcomes or treatment-related mortality in older patients treated with ide-cel, other studies have noted inferior survival in frailer populations treated with BCMA-directed CAR T-cells (63–66). Earlier use therefore requires consideration of fitness and the ability to tolerate treatment and recovery, alongside the expected benefit over available alternatives.

### 3.2 Bispecific antibodies

#### 3.2.1 From late-line therapy to earlier relapse

T-cell-redirecting bispecific antibodies have become an established treatment class in relapsed/refractory myeloma. These agents bind CD3 on T cells and a plasma cell antigen on myeloma cells, redirecting endogenous T cells toward malignant plasma cells and inducing T-cell-mediated cytotoxicity (67). Approved targets include BCMA and GPRC5D, while FcRH5 and trispecific constructs remain in development. Initial approvals were based on single-arm studies in heavily pretreated disease, but randomized phase 3 data now support use earlier in relapse.

Four bispecific antibodies are currently FDA-approved in RRMM, including teclistamab, talquetamab, elranatamab, and linvoseltamab. Teclistamab, the first approved bispecific antibody in myeloma, targets BCMA and CD3. In MajesTEC-1, teclistamab produced an ORR of 63.0%, with CR or better in 39.4%, in a heavily pretreated population. MRD negativity was achieved in 26.7% overall and in 46% of patients achieving CR or better (68). These results established that an off-the-shelf T-cell-redirecting therapy could produce deep responses without patient specific manufacturing.

Subsequent bispecific approvals expanded both the number of agents and the range of antigen targets. In MonumenTAL-1, talquetamab produced an ORR of 70% in patients with RRMM, establishing GPRC5D as an active non-BCMA target (69). The BCMA-directed antibodies elranatamab and linvoseltamab resulted in ORRs of 61% and 71% in MagnetisMM-3 (70) and LINKER-MM1 (71), respectively, with responses also observed in high-risk subgroups. Longer follow-up of LINKER-MM1 supported durability of these responses (72). Together, these single-arm studies established bispecific antibodies as an active late-line treatment class and provided the basis for evaluation earlier in the disease course.

Randomized early-relapse data now include teclistamab-daratumumab, teclistamab monotherapy, and talquetamab-based combinations. In the phase 3 MajesTEC-3 clinical trial, patients with RRMM after 1–3 prior lines of therapy were randomized to teclistamab-daratumumab or investigator’s choice of DPd or DVd (73). At a median follow-up of 34.5 months, teclistamab-daratumumab markedly improved PFS, with an estimated 36-month PFS of 83.4% compared with 29.7%. Responses were deeper, including higher CR or better (81.8% vs. 32.1%) and MRD negativity at 10^-5^ (58.4% vs. 17.1%). Estimated 36-month OS was also higher, 83.3% versus 65.0%. Time to worsening of symptoms was also delayed, supporting that the benefit was not restricted to laboratory or response-based endpoints.

Beyond its efficacy results, MajesTEC-3 is also important for its safety and supportive-care implications. CRS occurred in 60.1% of patients receiving teclistamab-daratumumab, was limited to grade 1 or 2 events, occurred mainly during step-up dosing, and did not lead to treatment discontinuation. ICANS was uncommon at 1.1%. Infections were more frequent with teclistamab-daratumumab, with grade 3/4 infections in 54.1% versus 43.4%, and fatal infections in 4.6% versus 1.4% compared with DPd or DVd. Most infection-related deaths occurred in the first 6 months and before reinforced immunoglobulin-replacement guidance was implemented. While the efficacy results are impressive, the infection data highlight that infection prevention and immunoglobulin monitoring are central to delivery of this regimen.

MajesTEC-9 evaluated teclistamab monotherapy in the early-relapse setting. In this phase 3 trial, patients with one to three prior lines, including prior anti-CD38 monoclonal antibody and lenalidomide exposure, were randomized to teclistamab or investigator’s choice of PVd or Kd. At a median follow-up of 17.3 months, teclistamab improved estimated 18-month PFS from 26.9% to 69.8%, increased CR or better from 16.8% to 65.9%, and improved estimated 18-month OS from 68.6% to 79.2% (74). Grade 3/4 infections remained common, occurring in 41.6% with teclistamab versus 29.0% with PVd or Kd, with grade 5 adverse events in 6.5% versus 3.5%.

MonumenTAL-3 extends randomized early-relapse evidence to GPRC5D-directed bispecific therapy. In this phase 3 trial, patients with RRMM after at least one prior line were randomized to talquetamab-daratumumab-pomalidomide, talquetamab-daratumumab, or DPd. At a median follow-up of 24.6 months, 24-month PFS was 81.3% with Tal-DP and 77.6% with Tal-D, compared with 51.2% with DPd. Responses were also deeper, with CR or better in 71.1%, 69.0%, and 34.5%, respectively, and MRD-negative CR at 10^–5^ in 52.3%, 46.3%, and 15.9%. Overall survival estimates numerically favored the talquetamab-containing arms, although the prespecified significance boundary for OS was not crossed at this interim analysis. In the talquetamab-containing arms, CRS was common but mainly grade 1 or 2, ICANS was uncommon, and GPRC5D-related toxicities included taste changes, skin and nail events, weight loss, and ataxia or balance disorders (75).

With randomized early-relapse data now available for both teclistamab- and talquetamab-based strategies, selection among bispecific antibodies in the early-line setting should be individualized because no trial has directly compared these agents head-to-head. In practice, choice is driven by many factors, including regulatory indication, target antigen, disease tempo, comorbidities, anticipated CAR T-cell therapy, and patient preference. Teclistamab has the most mature randomized evidence in earlier relapse, including both monotherapy and combination data, while data for other bispecific antibodies continue to evolve. Available real-world and indirect comparisons suggest that toxicity profiles may differ by target, but evidence is largely retrospective, often reported in abstract form only, subject to confounding by patient selection, prior therapy, supportive care, and toxicity reporting. Retrospective comparisons suggest higher rates of CRS with talquetamab than with BCMA-directed bispecific antibodies (76) (77). Analyses of infectious complications have also suggested greater infection risk with BCMA-directed bispecific antibodies than with GPRC5D-directed therapy, although confounding by patient selection, steroid exposure, prophylaxis, and prior therapy remains difficult to exclude (78, 79). Nonetheless, CRS and infections remain risks across the class, and prophylactic approaches, including tocilizumab-based CRS mitigation and intravenous immunoglobulin (IVIG)-based infection prevention may reduce clinically significant toxicity, although optimal patient selection, timing, and duration remain unsettled (80, 81). Infection mitigation is particularly important given the persistent risk throughout bispecific treatment unlike CRS and ICANS which usually occur closer to bispecific initiation. Talquetamab also carries distinct GPRC5D-related oral, taste, skin, and nail toxicities, which are often low grade but can be functionally important, particularly when therapy is intended to be continuous.

As BCMA-directed CAR T-cell therapy and bispecific antibodies move earlier, sequencing becomes increasingly important. Prior BCMA-directed therapy may reduce the activity of subsequent BCMA-directed treatment through antigen loss, antigen modulation, T-cell dysfunction or selection of resistant clones (82, 83). Conversely, non-BCMA targeting with talquetamab may preserve BCMA as a later target, but at the cost of a distinct toxicity profile. An important unanswered question is therefore not simply which bispecific antibody has the highest response rate, but which antigen sequence gives patients the best chance of receiving and benefiting from multiple effective immunotherapies over time. Prospective sequencing studies and large real-world datasets will be essential.

#### 3.2.2 Current studies investigating bispecific antibody expansion to frontline use

Frontline bispecific antibody studies are testing their use in various settings, including induction, consolidation, MRD-directed intensification, and maintenance.

MajesTEC-7 (NCT05552222*)* is a phase 3 randomized trial comparing teclistamab, daratumumab, and lenalidomide (Tec-DR), and talquetamab, daratumumab, and lenalidomide (Tal-DR) to DRd in transplant-ineligible patients or those not intended for upfront ASCT. Early safety run-in data, published in abstract form, with Tec-DR showed an ORR of 92.3%, with CR or better in 73.1% at a median follow-up of 10.2 months (84). These findings are encouraging but come from a small, non-randomized cohort, and mature MajesTEC-7 results will clarify whether dexamethasone-sparing bispecific combinations improve on DRd while maintaining acceptable tolerability and treatment burden.

The IFM2021–01 TECLILLE (NCT05572229) provides preliminary single-arm frontline data, in abstract form, for teclistamab-based doublets in older, transplant-ineligible patients. In cohort A, teclistamab and daratumumab produced VGPR or better in 97% of patients. MRD negativity rate at 10^–6^ at 6 months was 51% in the intention-to-treat population and 100% in the 21 evaluable patients, with no progression or death reported at a median follow-up of 7.6 months (85). Teclistamab and lenalidomide are being evaluated in a separate cohort.

In transplant-eligible patients, the most mature published frontline data come from MajesTEC-5 (NCT05695508), a phase 2 study evaluating teclistamab-based induction with daratumumab and lenalidomide, with or without bortezomib, followed by ASCT. In a pooled analysis, ORR was 100% and MRD-negative CR at 10^-5^ was achieved in 45 of 49 patients (91.8%) by the premaintenance time point. Toxicity was common, with grade 3 or 4 adverse events in 91.8%, grade 3 or 4 infections in 36.7%, and CRS in 67.3%, although CRS was limited to grade 1 or 2 events and no grade 5 treatment-emergent adverse events were reported (86). Whether these deep early responses translate into better long-term disease control than contemporary anti-CD38 quadruplet induction is unknown.

Several other studies are testing BCMA-directed bispecific therapy in more selective frontline roles. IMMUNOPLANT (NCT06376526) is evaluating abbreviated linvoseltamab consolidation in patients who remain MRD-positive after standard triplet or quadruplet therapy. Early data published in abstract form reported all 14 evaluable patients among 19 enrolled achieved MRD negativity after four cycles, although median follow-up was only 3.9 months (87). LINKER-MM4 (NCT05828511) evaluates linvoseltamab monotherapy during induction and consolidation with preliminary activity reported (88). Longer follow-up will be needed to determine whether these responses are sustained.

Differences in patient populations, assay sensitivity and timing of assessment nonetheless limit cross-study comparison of these frontline response rates. MRD negativity after induction is not directly comparable with MRD negativity achieved after ASCT or additional consolidation and maintenance. Likewise, high rates among evaluable patients may not reflect the proportion of all enrolled patients who achieved MRD negativity, particularly when assessments are missing (85, 86).

Other transplant-eligible studies are evaluating bispecific antibodies after induction or after ASCT. TALTEC (NCT06505369) is studying sequential talquetamab and teclistamab consolidation after D-VRd. GEM-TECTAL (NCT05849610) is evaluating teclistamab-daratumumab intensification after Dara-VRd in high-risk newly diagnosed myeloma, with talquetamab-daratumumab used for patients with persistent MRD positivity or those achieving less than CR. MajesTEC-4/EMN30 (NCT05243797) is evaluating post-ASCT maintenance with teclistamab-lenalidomide, teclistamab alone, or lenalidomide alone (89).

Bispecific antibodies are also being studied together with CAR T-cell therapy. MonumenTAL-8 (NCT06550895) is evaluating talquetamab with cilta-cel in high-risk myeloma, including talquetamab as bridging before cilta-cel and as consolidation after cilta-cel. The aMMbition study (NCT06577025) is evaluating frontline cilta-cel sequencing strategies in newly diagnosed myeloma, including combinations with teclistamab-daratumumab and talquetamab-daratumumab (67). These studies should provide early data on whether bispecific antibodies can be integrated before or after CAR T-cell therapy.

Bispecific therapy is also being evaluated in high-risk smoldering myeloma, where treatment benefit must be balanced against the risk of intervening in patients who are asymptomatic. LINKER-SMM1 (NCT05955508) is studying linvoseltamab monotherapy and reported 100% ORR after one cycle in early results (90). REVIVE (NCT06100237) is evaluating teclistamab-daratumumab and talquetamab-daratumumab, while Immuno-PRISM (NCT05469893) is comparing teclistamab with Rd. Conference abstracts have reported deep responses, including high rates of MRD negativity, but longer follow-up is needed to determine whether early bispecific therapy delays progression with acceptable toxicity and treatment burden (91, 92).

#### 3.2.3 Novel bispecific antibody targets

Beyond the approved BCMA- and GPRC5D-directed bispecific antibodies, several investigational agents are attempting to refine target engagement, dosing, or antigen selection. Etentamig is a BCMA x CD3 bispecific antibody with bivalent BCMA binding, low-affinity CD3 binding, and an extended half-life. This design is intended to preserve anti-myeloma activity while reducing CRS and allowing less frequent dosing (93). Published etentamig data remain limited to relapsed/refractory myeloma. In newly diagnosed disease, a phase 1/2 platform study is evaluating etentamig plus daratumumab and lenalidomide in transplant-ineligible patients, as well as etentamig alone or with lenalidomide as maintenance in transplant-eligible patients (NCT06892522). A separate phase 2/3 trial is comparing etentamig plus daratumumab with DRd in transplant-ineligible patients (NCT07095452) (94).

Cevostamab is an investigational FcRH5 × CD3 bispecific antibody. Fc receptor-homolog 5 (FcRH5) is a B-lineage restricted membrane protein with near universal expression on multiple myeloma cells, making it an attractive non-BCMA and non-GPRC5D target (95). Several studies are evaluating cevostamab in relapsed/refractory disease, including after prior BCMA-directed therapy. The ongoing phase 3 CEVOLUTION trial (NCT07555938) is comparing cevostamab plus pomalidomide and dexamethasone with investigator’s choice of standard pomalidomide-based therapy in patients who have received one to three prior lines and have been exposed to lenalidomide and an anti-CD38 monoclonal antibody.

Ramantamig (JNJ-79635322) is an investigational trispecific antibody targeting BCMA, GPRC5D, and CD3 (96). Its dual-antigen design may reduce dependence on uniform expression of either BCMA or GPRC5D, although whether this improves the depth or durability of response remains to be seen. A phase 1 study is evaluating ramantamig-based combinations in both newly diagnosed myeloma and after one to three prior lines of therapy (NCT06768489). In addition, the phase 3 TRIlogy-5 trial (NCT07518186) compares ramantamig with teclistamab in patients who have received one to three prior lines, including lenalidomide and an anti-CD38 antibody. Several studies include patients with prior BCMA exposure, an increasingly relevant population as BCMA-directed therapy moves earlier. Selected early-line studies of novel bispecific and trispecific antibodies are summarized in **Table 2**.

**Table 2:** Selected investigational bispecific and trispecific strategies in frontline and early-line multiple myeloma.

Clinical setting	Trial	Product/target	Phase and design	Key population	Treatment strategy/comparator	Primary endpoints and available results
Frontline induction	**NCT07095452**	Etentamig (ABBV-383); BCMAxCD3 bispecific	Phase II/III; multicenter; randomized; open-label	NDMM, transplant-ineligible	Etentamig + daratumumab vs. DRd	Primary endpoints: Clinical activity (ORR, CR, VGPR, PR), MRD negative (10^–5^) CR rate, PFS, incidence of AEs Available results: Ongoing study; data not yet available (94)
Frontline induction and early relapse	**NCT06768489**	Ramantamig (JNJ-79635322); BCMAxGPRC5DxCD3 trispecific	Phase I; multicohort combination study	Includes NDMM and early RRMM after 1–3 prior lines of therapy	Ramantamig with daratumumab ± lenalidomide or pomalidomide	Primary endpoints: Number of participants with dose-limiting toxicity, AEs by severity, and clinically significant lab abnormalities Available results: Ongoing study; data not yet available
Frontline induction	**NCT06892522**	Etentamig (ABBV-383); BCMAxCD3 bispecific	Phase I/II; open-label; randomized; platform study	Population varies by cohort and regimen, includes both NDMM and RRMM	Etentamig monotherapy or in combination with daratumumab, lenalidomide, dexamethasone, and/or carfilzomib	Primary endpoints: Incidence of AEs, dose-limiting toxicities of Etentamig monotherapy and combinations Available results: Ongoing study; data not yet available
Post-induction, response-adapted consolidation	**IMMUNOPLANT** **NCT06376526**	Linvoseltamab; BCMAxCD3 bispecific	Phase II; single-arm	NDMM patients remaining MRD-positive after initial standard combination therapy	Fixed-duration linvoseltamab consolidation	Primary endpoints: MRD negativity rate (10^–5^ by flow cytometry and 10^–6^ by NGS clonoSEQ) to determine MRD conversion rate after 4–6 cycles Available results: Early results show 100% conversion to MRD negativity after 4 cycles (n=14) by both NGS clonoSEQ and flow cytometry (87).
Frontline consolidation/maintenance	**FASTER NCT06974786**	Elranatamab; BCMAxCD3 bispecific	Phase II; randomized; open-label; response-adaptive	NDMM, transplant-eligible, ≥PR after 16–24 weeks of PI + lenalidomide + anti-CD38 induction	Elranatamab-daratumumab consolidation and maintenance vs. ASCT followed by lenalidomide-daratumumab maintenance, with MRD-guided sequential intensification	Primary endpoints: MRD negativity rate (10^–5^) after initial maintenance completion Available results: Ongoing study; data not yet available
Frontline/post-induction transition	**NCT06932562**	Linvoseltamab; BCMAxCD3 bispecific	Phase III; randomized; open-label	NDMM, transplant-ineligible	DRd induction followed by linvoseltamab vs. continued DRd	Primary endpoints: MRD negative (10^–5^) CR rate, PFS Available results: Ongoing study; data not yet available
Early relapse	**CEVOLUTION** **NCT07555938**	Cevostamab; FcRH5xCD3 bispecific	Phase III; randomized; open-label; multicenter	RRMM after 1–3 prior lines, including prior anti-CD38 antibody and lenalidomide	Cevostamab-pomalidomide-dexamethasone combination therapy vs. standard-of-care therapy	Primary endpoints: MRD negative CR rate, PFS Available results: Ongoing study; data not yet available
Early relapse	**TRIlogy-5** **NCT07518186**	Ramantamig (JNJ-79635322); BCMAxGPRC5DxCD3 trispecific	Phase III; randomized	RRMM after 1–3 prior lines, including prior anti-CD38 antibody and lenalidomide	Ramantamig vs. teclistamab	Primary endpoints: ≥CR rate, PFS Available results: Ongoing study; data not yet available

AE, adverse event; ASCT, autologous stem-cell transplantation; BCMA, B-cell maturation antigen; CD3, cluster of differentiation 3; CR, complete response; DRd, daratumumab-lenalidomide-dexamethasone; DKd, daratumumab-carfilzomib-dexamethasone; DPd, daratumumab-pomalidomide-dexamethasone; D-VRd, daratumumab-bortezomib-lenalidomide-dexamethasone; FcRH5, Fc receptor-homlog 5; GPRC5D, G protein-coupled receptor class C; IMiD, immunomodulatory drug; Kd, carfilzomib-dexamethasone; MM, multiple myeloma; MRD, measurable residual disease; NDMM, newly diagnosed multiple myeloma; ORR, overall response rate; PR, partial response; PVd, pomalidomide-bortezomib-dexamethasone; RRMM, relapsed/refractory multiple myeloma; VGPR, very good partial response.

Bolded items indicate clinical trial names and their corresponding NCT identification numbers.

#### 3.2.4 Potential challenges to expanding bispecific antibodies to frontline use

The main logistical advantage of bispecific antibodies over CAR T-cell therapy is immediate availability, since they do not require leukapheresis and individualized manufacturing. This may be particularly important for patients with rapidly progressive disease or limited access to centers offering cellular therapy. However, bispecific antibodies have their own operational challenges. Treatment initiation requires step-up dosing and CRS monitoring, while ongoing therapy requires infection prevention planning, pharmacy coordination, urgent assessment pathways, and access to inpatient care when needed.

##### 3.2.4.1 Need for initial step-up dosing

Early real-world experience with teclistamab illustrates that initiation often required inpatient resources, although practice continues to evolve. In a US claims analysis of 99 patients with RRMM who completed teclistamab step-up dosing, 87.9% did so during a single inpatient admission, with a median hospital stay of 8 days. Only 8.1% were treated using a hybrid inpatient and outpatient model, and only 4% completed step-up dosing entirely as outpatient (97). As experience with dexamethasone and prophylactic tocilizumab for CRS management and prevention, as well as with outpatient bispecific administration, grows, the logistical requirements will adjust accordingly (80, 98, 99). Nonetheless, broader frontline use would still require reliable pathways for toxicity monitoring and supportive care outside highly specialized centers.

##### 3.2.4.2 Risk of infections and infection-related mortality

Infection prevention is likely to be a major determinant of whether bispecific antibodies can be delivered safely and sustainably in earlier lines. BCMA-directed bispecific antibodies can cause prolonged hypogammaglobulinemia and recurrent infections, and as such there is ongoing need for IVIG and infection surveillance. While primary IVIG prophylaxis has been associated with improved infection-free survival, the risk of infections still persists (100). Observational data suggest that immune recovery may take months after treatment discontinuation, with high-grade infections occurring during the first 6 months and some patients requiring IVIG support for up to 2.5 years (101). These measures may reduce complications but add cost and clinic time. This is particularly important in newly diagnosed myeloma, where bispecific regimens will be compared with established outpatient anti-CD38 containing triplets and quadruplets that already produce deep responses for many patients.

##### 3.2.4.3 Need for continuous dosing schedule

The optimal duration of bispecific therapy also remains unknown. Unlike a single CAR T-cell infusion, most bispecific antibody trials involved continued treatment until progression or intolerance. Fixed-duration and MRD-adapted approaches could reduce cumulative infection risk, treatment visits and costs, while potentially limiting prolonged T-cell stimulation (101–103). A central question for earlier-line trials is whether these advantages come at the cost of disease control or subsequent treatment options. IMMUNOPLANT is evaluating MRD-directed linvoseltamab consolidation for four cycles, extended to six cycles for patients with persistent MRD (NCT06376526). MajesTEC-5 employs teclistamab-based induction and post-ASCT consolidation, followed by teclistamab-daratumumab maintenance for up to 18 cycles (86). Because maintenance may continue after bispecific therapy ends, stopping the bispecific does not necessarily provide an interval off all anti-myeloma treatment.

##### 3.2.4.4 How do we optimize treatment sequencing?

Finally, sequencing will become more complex as CAR T-cell therapy and bispecific antibodies both move to earlier treatment lines. Prior BCMA-directed therapy could affect subsequent treatment through antigen modulation or loss, resistant clonal selection or impaired T-cell fitness. In a multicenter real-world analysis of ide-cel, prior BCMA-targeted therapy was associated with shorter median PFS (3.2 months versus 9 months) (104). Nevertheless, same target retreatment can remain active, with ORRs of 60% for ciltacel in CARTITUDE-2 cohort C and 52.5% for teclistamab in MajesTEC-1 cohort C (105, 106). Most sequencing data come from small, heavily pretreated cohorts with varied prior BCMA exposure, so their applicability to newly diagnosed or minimally pretreated patients remains uncertain.

## 4 The evolving role of autologous stem cell transplantation

ASCT remains a standard consolidation approach for transplant-eligible patients with newly diagnosed myeloma. In IFM-2009 and DETERMINATION, early ASCT prolonged PFS after triplet induction compared to non-transplant arms, although neither study showed a consistent OS advantage (107, 108). With modern anti-CD38 quadruplets, many patients achieve deep responses before transplant, and it remains to be elucidated who are the patients who still require ASCT in order to achieve maximal survival outcomes.

Anti-CD38 containing quadruplet induction trials with ASCT provide a very high benchmark for any strategy aiming to defer transplant. In PERSEUS, D-VRd followed by ASCT, consolidation, and daratumumab-lenalidomide maintenance produced a 48-month PFS of 84%, with sustained MRD negativity at 10^-5^ for at least 12 months in 65% of patients (5). GMMG-HD7 similarly showed high post-transplant MRD negativity with Isa-VRd (66% vs. 48% with RVd) and prolonged PFS (109).

MRD-driven management decisions might further refine the use of ASCT or the peri-transplant treatment strategy. In the MIDAS study, patients who were MRD-negative at 10^–5^ after Isa-KRd induction were assigned to ASCT or continued Isa-KRd consolidation. Pre-maintenance MRD negativity at 10^–6^ was similar between arms, 86% with ASCT versus 84% with continued Isa-KRd. With a relatively short median follow-up of 16.8 months, longer follow-up is needed to determine whether deferring ASCT preserves sustained MRD negativity and PFS (62). In the MASTER trial, MRD-adapted treatment cessation after D-KRd and ASCT was associated with a 27% cumulative incidence of MRD resurgence or progression at 12 months in patients with two or more high-risk cytogenetic abnormalities, compared with 0–4% in those with zero or one, despite similar initial rates of MRD negativity across risk groups (61). Future studies might show how early-line immunotherapy could be incorporated in such MRD-guided management strategies, either with or without ASCT.

CARTITUDE-6 will provide the most direct data on whether immediate ASCT can be deferred in favor of CAR T-cell therapy after quadruplet induction. The trial compares D-VRd followed by cilta-cel with D-VRd followed by ASCT, with lenalidomide maintenance in both arms. This study will shed light on the comparative efficacy and safety of these two modalities. ASCT has long-term follow-up, predictable toxicity, and low non-relapse mortality (< 1-2%) at experienced centers (110), whereas CAR T-cell therapy has different challenges related to manufacturing, access, acute and delayed toxicity, and less mature follow-up in newly diagnosed myeloma.

Bispecific antibodies may also affect how ASCT is used, but current data are earlier phase, and most studies are examining how they can be incorporated together with transplant rather than directly compared with it. In MajesTEC-5/GMMG-HD10, teclistamab-based induction yielded high rates of deep remissions without compromising stem cell collection (median yield 8.1 × 10^-6^/kg) (86). Phase 3 studies in the post-transplant setting, including MajesTEC-4 and MagnetisMM-7, are evaluating whether bispecific antibodies can improve the depth or durability of response after ASCT. If ASCT is deferred after induction, stem-cell collection should still be considered so that transplant remains available later.

Direct comparative data between bispecific antibodies and ASCT are not yet available, but future trials should help clarify their relative roles as consolidation. The ongoing phase 2 FASTER (NCT06974786) trial is comparing elranatamab-daratumumab consolidation and maintenance with ASCT followed by lenalidomide-daratumumab maintenance, while the planned phase 3 DETERMINATION 2 trial (NCT07624513) will randomize patients with high-risk or MRD-positive disease to either ASCT or eight cycles of linvoseltamab, followed in both arms by isatuximab-iberdomide maintenance. Potential approaches to incorporating immunotherapy into frontline treatment are summarized in **Figure 1**.

**Figure 1:**
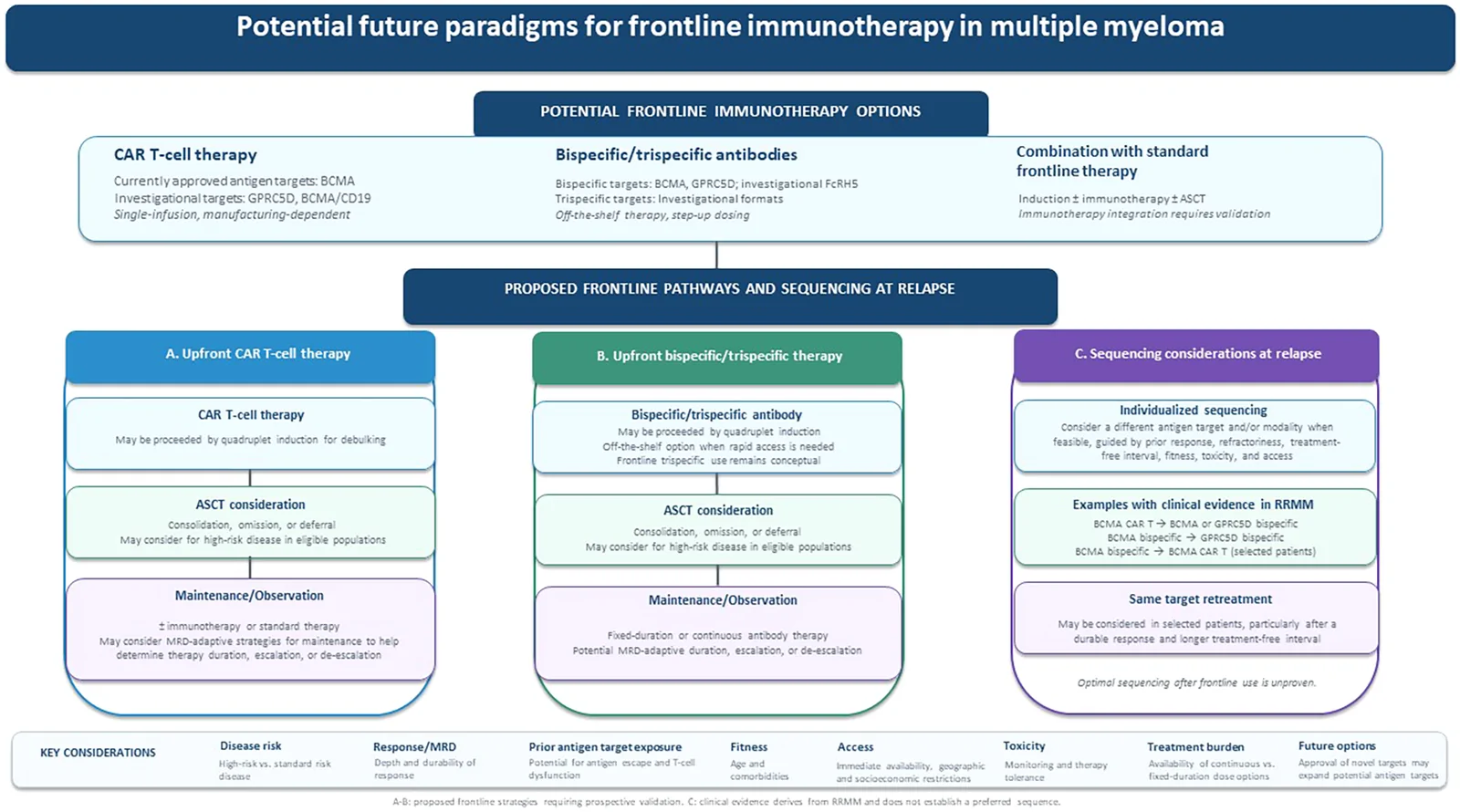
Proposed future framework for frontline immunotherapy and treatment sequencing at relapse in multiple myeloma. **(A, B)** depict conceptual frontline pathways, including potential integration of CAR T-cell therapy or bispecific/trispecific antibodies with induction, ASCT, and response-adapted maintenance or observation. These pathways require prospective validation, including the role and timing of ASCT and MRD-guided treatment adaptation. Frontline use of trispecific antibodies remains conceptual. Panel **(C)** summarizes individualized sequencing principles and illustrative sequences with clinical evidence in relapsed/refractory multiple myeloma (RRMM). Evidence of activity following prior immunotherapy does not establish comparative superiority or an optimal sequence, and its applicability after frontline exposure remains uncertain. ASCT, autologous stem-cell transplantation; BCMA, B-cell maturation antigen; CAR, chimeric antigen receptor; FcRH5, Fc receptor homolog 5; GPRC5D, G protein-coupled receptor class C group 5 member D; MRD, measurable residual disease; RRMM, relapsed/refractory multiple myeloma.

## 5 Conclusions and future directions

CAR T-cell therapies and T-cell–redirecting bispecific antibodies have moved from late-line salvage therapy into earlier relapse, where randomized trials have demonstrated substantial improvements over conventional regimens. Their role in newly diagnosed multiple myeloma remains uncertain. Although early studies have reported high response and MRD-negativity rates, these findings are based largely on selected populations, small cohorts and limited follow-up. These approaches must ultimately be compared with highly effective anti-CD38–based quadruplets, ASCT and maintenance strategies.

As immunotherapy moves earlier, optimal patient selection, treatment duration, and sequencing across targets and modalities will become increasingly important. Early use of BCMA-directed therapy may affect responses to subsequent BCMA-targeted treatment through mechanisms such as antigen modulation and altered T-cell fitness. Non-BCMA targets may preserve later therapeutic options but introduce distinct toxicities. Fixed-duration and MRD-adapted bispecific strategies could reduce toxicities and cumulative immune dysfunction, while also decreasing treatment burden and costs, but require large-scale prospective validation. Biomarkers reflecting T-cell fitness, antigen expression and immune microenvironment composition may help personalize immunotherapy strategies.

Ongoing and future trials will determine whether early-line immunotherapy can translate deep, sustained responses into improved long-term survival and prolonged treatment-free intervals while permitting immune recovery and preserving subsequent therapeutic options. A proposed framework for future frontline and early-line treatment sequencing in multiple myeloma is presented in **Figure 1**.

## References

[1] Kahlon N Bansal N Baddam S Rajput J Qureshi Z . Targeted therapeutics and U.S. population-level mortality trends in multiple myeloma: A SEER-based analysis from 1975 to 2023. Oncotarget. (2026) 17:216–25. doi: 10.18632/oncotarget.28877 42047296 PMC13249087

[2] Fonseca R Usmani SZ Mehra M Slavcev M He J Cote S . Frontline treatment patterns and attrition rates by subsequent lines of therapy in patients with newly diagnosed multiple myeloma. BMC Cancer. (2020) 20(1):1087. doi: 10.1186/s12885-020-07503-y 33172403 PMC7656738

[3] Rodríguez-Lobato LG de Daniel A Pereira A Fernández de Larrea C Tovar N Cibeira MT . Attrition rates and treatment outcomes in multiple myeloma: real-world data over a 40-year period. Blood Cancer J. (2025) 15(1):103. doi: 10.1038/s41408-025-01311-y PMC12106733 40419498

[4] van de Donk NWCJ Moreau P San-Miguel JF Mateos M-V Dimopoulos MA Zweegman S . Optimising T-cell immunotherapy in patients with multiple myeloma: practical considerations from the European Myeloma Network. Lancet Haematology. (2025) 12:e635–49. doi: 10.1016/s2352-3026(25)00117-6 40580975

[5] Sonneveld P Dimopoulos MA Boccadoro M Quach H Ho PJ Beksac M . Daratumumab, bortezomib, lenalidomide, and dexamethasone for multiple myeloma. N Engl J Med. (2024) 390:301–13. doi: 10.1056/nejmoa2312054 38084760

[6] Facon T Dimopoulos M-A Leleu XP Beksac M Pour L Hájek R . Isatuximab, bortezomib, lenalidomide, and dexamethasone for multiple myeloma. N Engl J Med. (2024) 391:1597–609. doi: 10.1056/nejmoa2400712 38832972

[7] Voorhees PM Kaufman JL Laubach J Sborov DW Reeves B Rodriguez C . Daratumumab, lenalidomide, bortezomib, and dexamethasone for transplant-eligible newly diagnosed multiple myeloma: the GRIFFIN trial. Blood. (2020) 136:936–45. doi: 10.1182/blood.2020005288 32325490 PMC7441167

[8] Leleu X Hulin C Lambert J Bobin A Perrot A Karlin L . Isatuximab, lenalidomide, dexamethasone and bortezomib in transplant-ineligible multiple myeloma: the randomized phase 3 BENEFIT trial. Nat Med. (2024) 30:2235–41. doi: 10.1038/s41591-024-03050-2 38830994 PMC11333283

[9] Cohen AD Raje N Fowler JA Mezzi K Scott EC Dhodapkar MV . How to train your T cells: overcoming immune dysfunction in multiple myeloma. Clin Cancer Res. (2020) 26:1541–54. doi: 10.1158/1078-0432.ccr-19-2111 31672768 PMC8176627

[10] Korst CLBM Tahri S Duetz C Bruins WSC de Jonge AV de Jong ME . The bone marrow NK-cell profile predicts MRD negativity in patients with multiple myeloma treated with daratumumab-based therapy. Blood. (2025) 145:3007–14. doi: 10.1182/blood.2024026455 40019438

[11] Maura F Boyle EM Coffey D Maclachlan K Gagler D Diamond B . Genomic and immune signatures predict clinical outcome in newly diagnosed multiple myeloma treated with immunotherapy regimens. Nat Cancer. (2023) 4:1660–74. doi: 10.1038/s43018-023-00657-1 37945755 PMC12065606

[12] Cortes-Selva D Perova T Skerget S Vishwamitra D Stein S Boominathan R . Correlation of immune fitness with response to teclistamab in relapsed/refractory multiple myeloma in the MajesTEC-1 study. Blood. (2024) 144:615–28. doi: 10.1182/blood.2023022823 38657201 PMC11347796

[13] Friedrich MJ Neri P Kehl N Michel J Steiger S Kilian M . The pre-existing T cell landscape determines the response to bispecific T cell engagers in multiple myeloma patients. Cancer Cell. (2023) 41:711–725.e6. doi: 10.1016/j.ccell.2023.02.008 36898378

[14] Verkleij CPM O’Neill CA Broekmans MEC Frerichs KA Bruins WSC Duetz C . T-cell characteristics impact response and resistance to T-cell–redirecting bispecific antibodies in multiple myeloma. Clin Cancer Res. (2024) 30:3006–22. doi: 10.1158/1078-0432.ccr-23-3333 38687588

[15] Chung DJ Pronschinske KB Shyer JA Sharma S Leung S Curran SA . T-cell exhaustion in multiple myeloma relapse after autotransplant: optimal timing of immunotherapy. Cancer Immunol Res. (2016) 4:61–71. doi: 10.1158/2326-6066.cir-15-0055 26464015 PMC4703436

[16] Suen H Brown R Yang S Weatherburn C Ho PJ Woodland N . Multiple myeloma causes clonal T-cell immunosenescence: identification of potential novel targets for promoting tumour immunity and implications for checkpoint blockade. Leukemia. (2016) 30:1716–24. doi: 10.1038/leu.2016.84 27102208

[17] Behsen AD Vandsemb EN Slørdahl TS Dybkær K Jakobsen MZ Kashif M . T cell phenotype and clonality changes in myeloma patients with short overall survival. JCI Insight. (2025) 10(11):e181096. doi: 10.1172/jci.insight.181096 40299757 PMC12220974

[18] Shasha C Glass DR Moelhman E Islas L Tian Y Chour T . Hallmarks of T-cell exhaustion and antigen experience are absent in multiple myeloma from diagnosis to maintenance therapy. Blood. (2025) 145:3113–23. doi: 10.1182/blood.2024025655 40163891 PMC12782984

[19] Foster KA Rees E Ainley L Laidler A Boyle EM Lee L . Tumour-intrinsic features shape T cell differentiation through precursor to symptomatic multiple myeloma. Nat Commun. (2026) 17(1):2400. doi: 10.1038/s41467-026-68718-4 41644568 PMC12982522

[20] Friedrich MJ Raab MS . When the silence speaks: T-cell dysfunction in myeloma. Blood. (2025) 145:3064–6. doi: 10.1182/blood.2025029262 40569640

[21] Ninkovic S Purton LE Harrison SJ Quach H . Multiplex immunohistochemistry elucidates increased distance between cytotoxic T cells and plasma cells in relapsed myeloma, and identifies Lag-3 as the most common checkpoint receptor on cytotoxic T cells of myeloma patients. Haematologica. (2023) 109(5):1487–500. doi: 10.3324/haematol.2023.283344 37855027 PMC11063850

[22] McCurdy A Mian H LeBlanc R Jimenez-Zepeda VH Su J Masih-Khan E . Correction: Redefining attrition in multiple myeloma (MM): a Canadian Myeloma Research Group (CMRG) analysis. Blood Cancer J. (2023) 13(1):129. doi: 10.1038/s41408-023-00888-6 37635183 PMC10460788

[23] Dhakal B Einsele H Schecter JM Deraedt W Lendvai N Slaughter A . Real-world treatment patterns and outcomes in relapsed/refractory multiple myeloma (1-3 prior lines): Flatiron database. Blood Adv. (2024) 8:5062–71. doi: 10.1182/bloodadvances.2024012640 39110988 PMC11459902

[24] Munshi NC Anderson LD Shah N Madduri D Berdeja J Lonial S . Idecabtagene vicleucel in relapsed and refractory multiple myeloma. N Engl J Med. (2021) 384:705–16. doi: 10.1056/nejmoa2024850 33626253

[25] Lin Y Martin TG Usmani SZ Berdeja JG Jakubowiak AJ Agha ME . CARTITUDE-1 final results: Phase 1b/2 study of ciltacabtagene autoleucel in heavily pretreated patients with relapsed/refractory multiple myeloma. J Clin Oncol. (2023) 41:8009. doi: 10.1016/s2152-2650(23)01433-7

[26] Jagannath S Martin TG Lin Y Cohen AD Raje N Htut M . Long-term (≥5-year) remission and survival after treatment with ciltacabtagene autoleucel in CARTITUDE-1 patients with relapsed/refractory multiple myeloma. J Clin Oncol. (2025) 43:2766–71. doi: 10.1200/jco-25-00760 40459151 PMC12393059

[27] Rodriguez-Otero P Ailawadhi S Arnulf B Patel K Cavo M Nooka AK . Ide-cel or standard regimens in relapsed and refractory multiple myeloma. N Engl J Med. (2023) 388:1002–14. doi: 10.1056/nejmoa2213614 36762851

[28] Ailawadhi S Arnulf B Patel K Cavo M Nooka AK Manier S . Ide-cel vs standard regimens in triple-class–exposed relapsed and refractory multiple myeloma: updated KarMMa-3 analyses. Blood. (2024) 144:2389–401. doi: 10.1182/blood.2024024582 39197072

[29] San-Miguel J Dhakal B Yong K Spencer A Anguille S Mateos M-V . Cilta-cel or standard care in lenalidomide-refractory multiple myeloma. N Engl J Med. (2023) 389:335–47. doi: 10.1056/nejmoa2303379 37272512

[30] Einsele H San-Miguel J Dhakal B Touzeau C Leleu X van de Donk NWCJ . Cilta-cel in lenalidomide-refractory multiple myeloma (CARTITUDE-4): an updated analysis including overall survival from an open-label, multicentre, randomised, phase 3 trial. Lancet Oncol. (2026) 27:254–68. doi: 10.1016/s1470-2045(25)00653-9 41519141

[31] Gagelmann N Einsele H Flossdorf S Smaili S Metzeler K Scheid C . Standard-of-care ciltacabtagene autoleucel in earlier versus later lines of therapy for relapsed or refractory multiple myeloma: a nationwide registry analysis. J Hematol Oncol. (2026) 19(1):46. doi: 10.1186/s13045-026-01806-6 42163304 PMC13307700

[32] Hansen DK Peres LC Dima D Richards A Shune L Afrough A . Comparison of standard-of-care idecabtagene vicleucel and ciltacabtagene autoleucel in relapsed/refractory multiple myeloma. J Clin Oncol. (2025) 43:1597–609. doi: 10.1200/jco-24-01730 39965175 PMC12037312

[33] Dytfeld D Dhakal B Agha M Manier S Delforge M Kuppens S . Bortezomib, lenalidomide and dexamethasone (VRd) followed by ciltacabtagene autoleucel versus vrd followed by lenalidomide and dexamethasone (Rd) maintenance in patients with newly diagnosed multiple myeloma not intended for transplant: A randomized, phase 3 study (CARTITUDE-5). Blood. (2021) 138:1835. doi: 10.1182/blood-2021-146210

[34] Boccadoro M San-Miguel J Suzuki K Van De Donk NWCJ Cook G Jakubowiak A . DVRd followed by ciltacabtagene autoleucel versus DVRd followed by ASCT in patients with newly diagnosed multiple myeloma who are transplant eligible: A randomized phase 3 study (<i>EM</i>agine/CARTITUDE-6). Blood. (2022) 140:4630–2. doi: 10.1182/blood-2022-157021

[35] Usmani SZ Berdeja JG Truppel-Hartmann A Casadebaig M-L Wortman-Vayn H Shelat SG . KarMMa-4: idecabtagene vicleucel (ide-cel, bb2121), a BCMA-directed CAR T-cell therapy, in high-risk newly diagnosed multiple myeloma. Blood. (2020) 136:18–9. doi: 10.1182/blood-2020-139009

[36] Yan W Du C Lv R Zou H Zhang S Yu T . Phase II study of BCMA chimeric antigen receptor T-cell therapy in patients with newly diagnosed multiple myeloma ineligible for or not proceeding to autologous stem-cell transplantation (CAREMM-001). J Clin Oncol. (2026) 44:992–1002. doi: 10.1200/jco-25-01969 41759038

[37] Chen B Shao X Jin Y Xu Y Shi Q Guan C . Efficacy and safety of a fully human BCMA CAR T-cell therapy for high-risk newly diagnosed transplant-ineligible multiple myeloma: Updated results from an open label, single-arm,phase 1 study(fumanba-2). Blood. (2025) 146:254. doi: 10.1182/blood-2025-254

[38] Nadeem O Cordas dos Santos DM Nikiforow S Bosch-Vilaseca A O’Donnell E Redd R . Ciltacabtagene autoleucel in high-risk smoldering multiple myeloma: the CAR-PRISM phase 2 trial. Nat Med. (2026) 32:2420–9. doi: 10.1038/s41591-026-04365-y 42010117 PMC13375535

[39] Bal S Htut M Berdeja J Anderson L Rossi A Gregory T . P33 | ARLOCABTAGENE AUTOLEUCEL, A GPRC5D-TARGETED CAR T-CELL THERAPY FOR PATIENTS WITH RELAPSED/REFRACTORY MULTIPLE MYELOMA: UPDATED PHASE 1 SAFETY AND EFFICACY RESULTS IN PATIENTS WITH 1–3 PRIOR REGIMENS. Haematologica. (2026) 111. doi: 10.3324/haematol.2026.s2.14112

[40] Lebel E Abdulgawad A Bramanti S Garate G Ho PJ Keränen M . Trial in progress: QUINTESSENTIAL-2—a phase 3 study of arlocabtagene autoleucel versus standard of care in adult patients with relapsed and refractory multiple myeloma (RRMM) exposed to lenalidomide. Blood. (2025) 146:2302. doi: 10.1182/blood-2025-2302

[41] Rodriguez-Otero P van de Donk NWCJ Pillarisetti K Cornax I Vishwamitra D Gray K . GPRC5D as a novel target for the treatment of multiple myeloma: a narrative review. Blood Cancer J. (2024) 14(1):24. doi: 10.1038/s41408-023-00966-9 38307865 PMC10837198

[42] Mailankody S Devlin SM Landa J Nath K Diamonte C Carstens EJ . GPRC5D-targeted CAR T cells for myeloma. N Engl J Med. (2022) 387:1196–206. doi: 10.1056/nejmoa2209900 36170501 PMC10309537

[43] van de Donk NWCJ Sonneveld P Einsele H . Dual-antigen–targeting T-cell immunotherapies in MM: circumventing tumor heterogeneity and preventing antigen escape. Blood. (2026) 147:2176–93. doi: 10.1182/blood.2025032536 41610427

[44] Mailankody S Mitra S Herrera K Ziccheddu B Cadzin B Eren OC . Concurrent administration of BCMA and GPRC5D chimeric antigen receptor (CAR) T cells in advanced multiple myeloma. Blood J. (2026) 148(17):2121–31. doi: 10.1182/blood.2025032122 42413007

[45] Liu J Fan X Peng L Liu J He H Qiang W . BCMA/CD19 dual-targeting CAR T-cell therapy in older patients with newly diagnosed multiple myeloma: a phase 1 study. Blood Adv. (2026) 10:4134–43. doi: 10.1182/bloodadvances.2025019036 41925575 PMC13310026

[46] Du J Qiang W Lu J Jia Y He H Liu J . A dual targeting BCMA and CD19 fastcar-t (GC012F/AZD0120) as first-line therapy for newly diagnosed multiple myeloma. Blood. (2025) 146:258. doi: 10.1182/blood-2025-258

[47] Patel KK Spooner C Giordana M Shuler T Myers D Mateos M-V . Anitocabtagene autoleucel (anito-cel) clinical trial manufacturing experience in patients with relapsed/refractory (RR) or newly diagnosed (ND) multiple myeloma (MM). J Clin Oncol. (2026) 44:2550. doi: 10.1200/jco.2026.44.16_suppl.2550

[48] Ge AY Feldman WB Kaiser MF Rejeski K Iacoboni G Narula G . Global access to commercial CAR T-cell therapies: a cross-sectional study of health technology assessment across the G20 countries. Blood. (2026) 147:1521–31. doi: 10.1182/blood.2025030872 41543441

[49] Kourelis T Bansal R Berdeja J Siegel D Patel K Mailankody S . Ethical challenges with multiple myeloma BCMA chimeric antigen receptor T cell slot allocation: A multi-institution experience. Transplant Cell Ther. (2023) 29:255–8. doi: 10.1016/j.jtct.2023.01.012 36681151 PMC10040426

[50] Brudno JN Maus MV Hinrichs CS . CAR T cells and T-cell therapies for cancer. JAMA. (2024) 332:1924. doi: 10.1001/jama.2024.19462 39495525 PMC11808657

[51] Delforge M Vekemans M-C Depaus J Meuleman N Van de Velde A Vande Broek I . Ciltacabtagene autoleucel for patients with triple-class exposed multiple myeloma: Adjusted comparison of CARTITUDE-1 patient outcomes versus real-world clinical practice. HemaSphere. (2022) 6:e813. doi: 10.1097/hs9.0000000000000813 36479545 PMC9722575

[52] Sidana S Patel KK Peres LC Bansal R Kocoglu MH Shune L . Safety and efficacy of standard-of-care ciltacabtagene autoleucel for relapsed/refractory multiple myeloma. Blood. (2025) 145:85–97. doi: 10.1182/blood.2024025945 39365257 PMC11952008

[53] Ailawadhi S Boytsov N Multani J Zhou Z Niehoff N Purser M . Gaps in access to chimeric antigen receptor T-cell (CAR-T) therapy post leukapheresis: Waiting time and post-leukapheresis treatment patterns in relapsed/refractory multiple myeloma (RRMM)—Real-world evidence from U.S. claims. J Clin Oncol. (2026) 44:7530. doi: 10.1200/jco.2026.44.16_suppl.753042852411

[54] Costa LJ Banerjee R Mian H Weisel K Bal S Derman BA . International myeloma working group immunotherapy committee recommendation on sequencing immunotherapy for treatment of multiple myeloma. Leukemia. (2025) 39:543–54. doi: 10.1038/s41375-024-02482-6 39870767 PMC11879857

[55] Swan D Madduri D Hocking J . CAR-T cell therapy in multiple myeloma: Current status and future challenges. Blood Cancer J. (2024) 14(1):206. doi: 10.1038/s41408-024-01191-8 39592597 PMC11599389

[56] Jiang N Yang Z Miao H Xing S Wang S Li N . Recent advances in universal chimeric antigen receptor T cell therapy. J Hematol Oncol. (2025) 18(1):82. doi: 10.1186/s13045-025-01737-8 40883768 PMC12398156

[57] Cordas dos Santos DM Tix T Shouval R Gafter-Gvili A Alberge J-B Cliff ERS . A systematic review and meta-analysis of nonrelapse mortality after CAR T cell therapy. Nat Med. (2024) 30:2667–78. doi: 10.1038/s41591-024-03084-6 38977912 PMC11765209

[58] Tix T Alhomoud M Shouval R Cliff ERS Perales M-A Cordas dos Santos DM . Second primary malignancies after CAR T-cell therapy: A systematic review and meta-analysis of 5,517 lymphoma and myeloma patients. Clin Cancer Res. (2024) 30:4690–700. doi: 10.1158/1078-0432.ccr-24-1798 39256908 PMC11546643

[59] Rejeski K Jain MD Shah NN Perales M-A Subklewe M . Immune effector cell-associated haematotoxicity after CAR T-cell therapy: From mechanism to management. Lancet Haematology. (2024) 11:e459–e70. doi: 10.1016/s2352-3026(24)00077-2 38734026 PMC12413773

[60] Hashmi H Sebastian T Rajeeve S Farzana T Firestone RS Jurgens EM . Functionally high-risk disease is associated with poor outcomes after late-line CAR T-cell therapy for multiple myeloma. Blood Cancer J. (2026) 16(1):74. doi: 10.1038/s41408-026-01494-y 41968163 PMC13181014

[61] Costa LJ Chhabra S Medvedova E Dholaria BR Schmidt TM Godby KN . Daratumumab, carfilzomib, lenalidomide, and dexamethasone with minimal residual disease response-adapted therapy in newly diagnosed multiple myeloma. J Clin Oncol. (2022) 40:2901–12. doi: 10.1200/jco.21.01935 34898239

[62] Perrot A Lambert J Hulin C Pieragostini A Karlin L Arnulf B . Measurable residual disease–guided therapy in newly diagnosed myeloma. N Engl J Med. (2025) 393:425–37. doi: 10.1056/nejmoa2505133 40459097 PMC13292240

[63] Akhtar OS Oloyede T Brazauskas R Afrough A Hashmi H Sidana S . Outcomes of older adults and frail patients receiving idecabtagene vicleucel: A CIBMTR study. Blood Adv. (2025) 9:1587–92. doi: 10.1182/bloodadvances.2024014970 39786391 PMC11986210

[64] Kalariya NM Hildebrandt MAT Hansen DK Sidana S Khouri J Ferreri CJ . Clinical outcomes after idecabtagene vicleucel in older patients with multiple myeloma: A multicenter real-world experience. Blood Adv. (2024) 8:4679–88. doi: 10.1182/bloodadvances.2024013540 39042903 PMC11402173

[65] Mian H Faisal MS Chen T Brazauskas R Oloyede T Ahmed N . Efficacy and safety of frail adults treated with ciltacabtagene autoleucel in the real-world: A CIBMTR analysis. Blood. (2025) 146:133. doi: 10.1182/blood-2025-133

[66] Davis JA Dima D Ahmed N DeJarnette S McGuirk J Jia X . Impact of frailty on outcomes after chimeric antigen receptor T cell therapy for patients with relapsed/refractory multiple myeloma. Transplant Cell Ther. (2024) 30:298–305. doi: 10.1016/j.jtct.2023.12.015 38142943

[67] Labanca C Martino EA Vigna E Bruzzese A Mendicino F Lucia E . Talquetamab in multiple myeloma: Efficacy, safety, and future directions. Eur J Haematol. (2024) 114:386–99. doi: 10.1111/ejh.14353 39604778 PMC11798766

[68] Moreau P Garfall AL van de Donk NWCJ Nahi H San-Miguel JF Oriol A . Teclistamab in relapsed or refractory multiple myeloma. N Engl J Med. (2022) 387:495–505. doi: 10.1056/nejmoa2203478 35661166 PMC10587778

[69] Chari A Minnema MC Berdeja JG Oriol A van de Donk NWCJ Rodríguez-Otero P . Talquetamab, a T-cell–redirecting GPRC5D bispecific antibody for multiple myeloma. N Engl J Med. (2022) 387:2232–44. doi: 10.1056/nejmoa2204591 36507686

[70] Lesokhin AM Tomasson MH Arnulf B Bahlis NJ Miles Prince H Niesvizky R . Elranatamab in relapsed or refractory multiple myeloma: Phase 2 MagnetisMM-3 trial results. Nat Med. (2023) 29:2259–67. doi: 10.1038/s41591-023-02528-9 37582952 PMC10504075

[71] Bumma N Richter J Jagannath S Lee HC Hoffman JE Suvannasankha A . Linvoseltamab for treatment of relapsed/refractory multiple myeloma. J Clin Oncol. (2024) 42:2702–12. doi: 10.1200/jco.24.01008 38879802 PMC11272139

[72] Lee HC Zonder JA Dhodapkar MV Jagannath S Hoffman JE Suvannasankha A . Linvoseltamab in patients with relapsed/refractory multiple myeloma in the LINKER-MM1 study: Longer follow-up and subgroup analyses. Clin Lymphoma Myeloma Leukemia. (2026) 26:e201–e12.e8. doi: 10.1016/j.clml.2025.11.004 41387038

[73] Costa LJ Bahlis NJ Perrot A Nooka AK Lu J Pawlyn C . Teclistamab plus daratumumab in relapsed or refractory multiple myeloma. N Engl J Med. (2026) 394:739–52. doi: 10.1056/nejmoa2514663 41363801 PMC13218738

[74] Touzeau C Mina R Quach H Hungria V Bhutani D Chen W . Teclistamab in multiple myeloma with one to three previous lines of therapy. N Engl J Med. (2026) 395:440–53. doi: 10.1056/nejmoa2603870 42212933

[75] Mina R Beksac M Rodríguez-Otero P Chen W Mateos M-V Li J . Talquetamab–daratumumab in relapsed or refractory myeloma. N Engl J Med. (2026) 395:671–83. doi: 10.1056/nejmoa2604657 42294841

[76] Khan AY Upadhyay L Fatima Z Safi D Safi SUD . Real-world comparison of anti-BCMA vs anti-GPRC5D BiTE therapy in relapsed/refractory multiple myeloma without prior CAR-T cell exposure. J Clin Oncol. (2025) 43:7542. doi: 10.1200/jco.2025.43.16_suppl.754242852411

[77] Khan AM Ali R Sangem A Rahim Z Niazi RK Parikh N . Comparative real-world outcomes with talquetamab and teclistamab in relapsed/refractory multiple myeloma: A TriNetX observational study. J Clin Oncol. (2026) 44(Suppl. 16):e19519. doi: 10.1200/jco.2026.44.16_suppl.e1951942852411

[78] Jourdes A Cellerin E Touzeau C Harel S Denis B Escure G . Characteristics and incidence of infections in patients with multiple myeloma treated by bispecific antibodies: A national retrospective study. Clin Microbiol Infection. (2024) 30:764–71. doi: 10.1016/j.cmi.2024.02.023 38432433

[79] Garcia Pleitez HJ Guevara N Das PJ Elhawi MA Aung WY Kamal SAF . Infection burden with BCMA versus GPRC5D-targeted bispecific antibodies in multiple myeloma: A systematic review and meta-analysis. J Clin Oncol. (2026) 44(Suppl. 16):e19511. doi: 10.1200/jco.2026.44.16_suppl.e1951142852411

[80] Kowalski A Lykon J Diamond B Coffey D Kaddoura M Maura F . Tocilizumab prophylaxis for patients with multiple myeloma treated with bispecific antibodies. Blood Adv. (2025) 9:4979–86. doi: 10.1182/bloodadvances.2025016911 40590849 PMC12514479

[81] Paulsen JS Slørdahl TS . To substitute or not? A systematic review of immunoglobulin replacement therapy in multiple myeloma patients treated with bispecific antibodies. Front Immunol. (2026) 16. doi: 10.3389/fimmu.2025.1722579 41601684 PMC12832799

[82] Lee H Ahn S Maity R Leblay N Ziccheddu B Truger M . Mechanisms of antigen escape from BCMA- or GPRC5D-targeted immunotherapies in multiple myeloma. Nat Med. (2023) 29:2295–306. doi: 10.1038/s41591-023-02491-5 37653344 PMC10504087

[83] van de Donk NWCJ Chari A Mateos MV . Mechanisms of resistance against T-cell engaging bispecific antibodies in multiple myeloma: Implications for novel treatment strategies. Lancet Haematology. (2024) 11:e693–707. doi: 10.1016/s2352-3026(24)00186-8 39033769

[84] Touzeau C Beksac M Terpos E Usmani SZ Krishnan AY Nijhof IS . Safety results from the phase 3 MajesTEC-7 study in patients (pts) with transplant ineligible/not intended newly diagnosed multiple myeloma (NDMM). J Clin Oncol. (2024) 42:7506. doi: 10.1200/jco.2024.42.16_suppl.750642852411

[85] Manier S Lambert J Macro M Chalopin T Dib M Rumpler A . A phase 2 study of teclistamab in combination with daratumumab in elderly patients with newly diagnosed multiple myeloma: The IFM2021-01 teclille trial, cohort a. Blood. (2025) 146:367. doi: 10.1182/blood-2025-367

[86] Raab MS Weinhold N Kortüm KM Krönke J Fenk R Weisel K . Teclistamab-based induction treatment in transplant-eligible, newly diagnosed multiple myeloma: A phase 2 trial. Nat Med. (2026) 32:2440–8. doi: 10.1038/s41591-026-04471-x 42350642 PMC13375549

[87] Kazandjian D Diamond B Hoffman J Pandey A Coffey D Kaddoura M . A phase 2 trial of abbreviated fixed-duration (Default 4 Cycles) linvoseltamab immuno-consolidation to deepen responses post newly diagnosed multiple myeloma combination therapy for minimal residual disease positivity (the IMMUNOPLANT Study). Blood. (2025) 146:248. doi: 10.1182/blood-2025-248

[88] Orlowski R Shah M Chakraborty R Fernandez-Abellán P Leleu X Oriol A . Safety and efficacy of linvoseltamab as a simplified monotherapy first-line regimen in NDMM: Initial results from the window of opportunity phase 1/2 LINKER-MM4 trial. Blood. (2025) 146:697. doi: 10.1182/blood-2025-697

[89] Zamagni E Boccadoro M Spencer A Delforge M Reece DE Gyula Szabo A . MajesTEC-4 (EMN30): A phase 3 trial of teclistamab + lenalidomide versus lenalidomide alone as maintenance therapy following autologous stem cell transplantation in patients with newly diagnosed multiple myeloma. Blood. (2022) 140:7289–91. doi: 10.1182/blood-2022-159756

[90] Rodríguez-Otero P Bargay Lleonart JJ Clavero ME Ribas P del Carmen Martínez-Chamorro M Sureda AM . Safety and efficacy of linvoseltamab (LINVO) in patients (pts) with high-risk smoldering multiple myeloma (HR-SMM): First results from the phase 2 LINKER-SMM1 trial. Clin Lymphoma Myeloma Leukemia. (2025) 25:S366–7. doi: 10.1016/s2152-2650(25)04011-x

[91] Landgren O Diamond B Pandey A Hoffman J Coffey D Kaddoura M . A phase 2 trial of teclistamab plus daratumumab combination therapy for high-risk smoldering multiple myeloma: First pre-planned analysis (the REVIVE Study). Blood. (2025) 146:5775. doi: 10.1182/blood-2025-5775

[92] Nadeem O Magidson S Midha S O'Donnell EK Hartley-Brown MA Sperling AS . Immuno-PRISM: A randomized phase II platform study of bispecific antibodies in high-risk smoldering myeloma. Blood. (2023) 142:206. doi: 10.1182/blood-2023-177954

[93] Baljevic M Voorhees PM Rodriguez Valdes C D'Souza A Chung A Lichtman EI . Long-term efficacy and safety of etentamig, a B-cell maturation antigen (BCMA) bispecific antibody in patients with relapsed/refractory multiple myeloma (RRMM). J Clin Oncol. (2025) 43:7527. doi: 10.1200/jco.2025.43.16_suppl.752742852411

[94] Manier S Puig N Leleu X Oriol A Hulin C García-Sanz R . A phase 2/3, multicenter, randomized, open-label study evaluating the efficacy and safety of etentamig and daratumumab compared to daratumumab, lenalidomide, and dexamethasone in frailer transplant-ineligible patients with newly diagnosed multiple myeloma. Blood. (2025) 146:2288. doi: 10.1182/blood-2025-2288

[95] Richter J Thomas SK Krishnan AY Laubach JP Cohen AD Trudel S . Cevostamab in patients with heavily pretreated relapsed/refractory multiple myeloma (RRMM): Updated results from an ongoing phase I study demonstrate clinically meaningful activity and manageable safety and inform the doses and regimen for combination studies. Blood. (2024) 144:1021. doi: 10.1182/blood-2024-199542

[96] Pillarisetti K Yang D Luistro L Yao J Smith M Vulfson P . Ramantamig (JNJ-79635322), a novel T-cell-engaging trispecific antibody targeting BCMA, GPRC5D, and CD3, in multiple myeloma models. Blood. (2026) 147:834–47. doi: 10.1182/blood.2025030027 41100731 PMC13000934

[97] Banerjee R Kim N Kohli M Hester L Achter E Fowler J . Evolving real-world characteristics and step-up dosing among early initiators of teclistamab for multiple myeloma - a national all-payer claims database study. Blood. (2023) 142:5087. doi: 10.1182/blood-2023-178908

[98] Davis JA Snyder J Rice M Moore DC Cahoon C Julian K . Dexamethasone for the management of CRS related to teclistamab in patients with relapsed/refractory multiple myeloma. Blood Cancer J. (2025) 15(1):32. doi: 10.1038/s41408-025-01222-y 40038247 PMC11880428

[99] Garfall AL Banerjee R Frenzel L Khandanpour C Lin Y Ottoni E . A roadmap to implementing outpatient administration of bispecific antibodies in multiple myeloma. Front Oncol. (2025) 15. doi: 10.3389/fonc.2025.1630146 40809030 PMC12343214

[100] Mohan M Szabo A Cheruvalath H Clennon A Bhatlapenumarthi V Patwari A . Effect of intravenous immunoglobulin (IVIG) supplementation on infection-free survival in recipients of BCMA-directed bispecific antibody therapy for multiple myeloma. Blood Cancer J. (2025) 15(1):74. doi: 10.1038/s41408-025-01282-0 40268898 PMC12018942

[101] Vega G Morillo D Riedhammer C Smits F Bag A Julian K . Sustained disease remission after a limited duration of bispecific antibody therapy in patients with multiple myeloma. HemaSphere. (2026) 10(6):e70396. doi: 10.1002/hem3.70396 42261378 PMC13242627

[102] Razzo B Chakraborty R Shaikh H Fiannaca L Girgis S Le S . Interim analysis of LimiTec, a prospective trial of limited-duration teclistamab for relapsed/refractory multiple myeloma. Blood. (2025) 146:5814. doi: 10.1182/blood-2025-5814

[103] Lesokhin AM Richter J Trudel S Cohen AD Spencer A Forsberg PA . Enduring responses after 1-year, fixed-duration cevostamab therapy in patients with relapsed/refractory multiple myeloma: Early experience from a phase I study. Blood. (2022) 140:4415–7. doi: 10.1182/blood-2022-157547

[104] Ferreri CJ Hildebrandt MAT Hashmi H Shune LO McGuirk JP Sborov DW . Real-world experience of patients with multiple myeloma receiving ide-cel after a prior BCMA-targeted therapy. Blood Cancer J. (2023) 13(1):117. doi: 10.1038/s41408-023-00886-8 37558706 PMC10412575

[105] Cohen AD Mateos M-V Cohen YC Rodriguez-Otero P Paiva B van de Donk NWCJ . Efficacy and safety of cilta-cel in patients with progressive multiple myeloma after exposure to other BCMA-targeting agents. Blood. (2023) 141:219–30. doi: 10.1182/blood.2022015526 36095849 PMC10562529

[106] Touzeau C Krishnan AY Moreau P Perrot A Usmani SZ Manier S . Efficacy and safety of teclistamab in patients with relapsed/refractory multiple myeloma after BCMA-targeting therapies. Blood. (2024) 144:2375–88. doi: 10.1182/blood.2023023616 39172760

[107] Attal M Lauwers-Cances V Hulin C Leleu X Caillot D Escoffre M . Lenalidomide, bortezomib, and dexamethasone with transplantation for myeloma. N Engl J Med. (2017) 376:1311–20. doi: 10.1056/nejmoa1611750 28379796 PMC6201242

[108] Richardson PG Jacobus SJ Weller EA Hassoun H Lonial S Raje NS . Triplet therapy, transplantation, and maintenance until progression in myeloma. N Engl J Med. (2022) 387:132–47. doi: 10.1056/nejmoa2204925 35660812 PMC10040899

[109] Mai EK Bertsch U Pozek E Fenk R Besemer B Hanoun C . Isatuximab, lenalidomide, bortezomib, and dexamethasone induction therapy for transplant-eligible newly diagnosed multiple myeloma: Final part 1 analysis of the GMMG-HD7 trial. J Clin Oncol. (2025) 43:1279–88. doi: 10.1200/jco-24-02266 39652594 PMC11974620

[110] Pasvolsky O Marcoux C Dai J Milton DR Tanner MR Syed N . Trends in outcomes after upfront autologous transplant for multiple myeloma over three decades. Transplant Cell Ther. (2024) 30:772.e1–.e11. doi: 10.1016/j.jtct.2024.06.001 38852784 PMC12412427

